# Dysbiotic Fecal Microbiome in HIV-1 Infected Individuals in Ghana

**DOI:** 10.3389/fcimb.2021.646467

**Published:** 2021-05-18

**Authors:** Prince Kofi Parbie, Taketoshi Mizutani, Aya Ishizaka, Ai Kawana-Tachikawa, Lucky Ronald Runtuwene, Sayuri Seki, Christopher Zaab-Yen Abana, Dennis Kushitor, Evelyn Yayra Bonney, Sampson Badu Ofori, Satoshi Uematsu, Seiya Imoto, Yasumasa Kimura, Hiroshi Kiyono, Koichi Ishikawa, William Kwabena Ampofo, Tetsuro Matano

**Affiliations:** ^1^ AIDS Research Center, National Institute of Infectious Diseases, Tokyo, Japan; ^2^ Noguchi Memorial Institute for Medical Research, University of Ghana, Accra, Ghana; ^3^ Joint Research Center for Human Retrovirus Infection, Kumamoto University, Kumamoto, Japan; ^4^ The Institute of Medical Science, The University of Tokyo, Tokyo, Japan; ^5^ Department of Internal Medicine, Regional Hospital Koforidua, Ghana Health Service, Koforidua, Ghana; ^6^ Department of Immunology and Genomics, Osaka City University Graduate School of Medicine, Osaka, Japan; ^7^ Collaborative Research Institute for Innovative Microbiology, The University of Tokyo, Tokyo, Japan; ^8^ Institute for Global Prominent Research, Graduate School of Medicine, Chiba University, Chiba, Japan; ^9^ Chiba University-University of California San Diego Center for Mucosal Immunology, Allergy and Vaccines (cMAV), Department of Medicine, University of California San Diego, San Diego, CA, United States

**Keywords:** HIV-1, gut microbiome, dysbiosis, sub‐Saharan Africa, Ghana

## Abstract

HIV-1 infected individuals under antiretroviral therapy can control viremia but often develop non-AIDS diseases such as cardiovascular and metabolic disorders. Gut microbiome dysbiosis has been indicated to be associated with progression of these diseases. Analyses of gut/fecal microbiome in individual regions are important for our understanding of pathogenesis in HIV-1 infections. However, data on gut/fecal microbiome has not yet been accumulated in West Africa. In the present study, we examined fecal microbiome compositions in HIV-1 infected adults in Ghana, where approximately two-thirds of infected adults are females. In a cross-sectional case-control study, age- and gender-matched HIV-1 infected adults (HIV+; n = 55) and seronegative controls (HIV-; n = 55) were enrolled. Alpha diversity of fecal microbiome in HIV+ was significantly reduced compared to HIV- and associated with CD4 counts. HIV+ showed reduction in varieties of bacteria including *Faecalibacterium*, the most abundant in seronegative controls, but enrichment of *Proteobacteria*. Ghanaian HIV+ exhibited enrichment of *Dorea* and *Blautia*; bacteria groups whose depletion has been reported in HIV-1 infected individuals in several other cohorts. Furthermore, HIV+ in our cohort exhibited a depletion of *Prevotella*, a genus whose enrichment has recently been shown in men having sex with men (MSM) regardless of HIV-1 status. The present study revealed the characteristics of dysbiotic fecal microbiome in HIV-1 infected adults in Ghana, a representative of West African populations.

## Introduction

Antiretroviral therapy (ART) can inhibit HIV-1 replication and prevent AIDS progression but is not able to eliminate the viruses in HIV-1 infected individuals (Dieffenbach and Fauci, 2011). HIV-1 infected individuals on ART are likely to develop non-AIDS diseases such as cardiovascular and metabolic disorders (Antiretroviral Therapy Cohort Collaboration, 2010). Gut dysbiosis in HIV-1 infected individuals has been indicated to be associated with progression of these non-AIDS diseases (Vujkovic-Cvijin et al., 2013; Nwosu et al., 2014; West et al., 2015; Kehrmann et al., 2019). Severe gastrointestinal barrier impairment resulting in translocation of microbial components from intestinal mucosa into blood stream during the acute phase of HIV-1 infection has been reported as a major driver of systemic inflammation and immune activation with subsequent detrimental disease outcomes (Brenchley et al., 2006; Marchetti et al., 2011; Sandler and Douek, 2012; Mudd and Brenchley, 2016). Circulating lipopolysaccharide (LPS) levels are considered to be a strong predictor of disease progression in HIV-1 infection (Marchetti et al., 2011), and association of circulating microbial product levels with systemic immune activation has been indicated in simian immunodeficiency virus (SIV) infected macaques as well as HIV-1 infected individuals (Brenchley et al., 2006).

Gut microbiome composition is different all over the world, and individual populations show varieties of biological interaction between host factors and microbiome. For instance, it has been suggested that diet and gut microbial compositions are determinant for higher risk of colorectal carcinogenesis in African Americans compared to native Africans and Caucasians (O’Keefe et al., 2007). On the other hand, influence of host genetics (HLA-B27 and HLA-DRB1) on gut microbial dysbiosis has been indicated in ankylosing spondylitis and rheumatoid arthritis (Gill et al., 2018; Asquith et al., 2019). Analysis of gut/fecal microbiome is thus important for our understanding of pathogenesis in HIV-1 infections in individual regions. Therapeutic interventions targeting gut microbiota have currently been explored (Irvine et al., 2011; Klatt et al., 2013; D’Ettorre et al., 2015; Ortiz et al., 2016), but lacking in data on gut/fecal microbiome may be an obstacle for application of these attempts. However, studies on gut microbiome in people living with HIV (PLWH) in sub-Saharan Africa, a population with the vast majority of PLWH, are limited (Gootenberg et al., 2017). Indeed, reports comparing microbial compositions of HIV-1 infected and uninfected groups adequately matched on age, gender, and residence are quite limited. To address the issues described above, we started analyzing fecal microbiome in HIV-1 infected individuals in Ghana, West Africa, where data on enteric microbiome has not yet been accumulated. Approximately two-thirds of HIV-1-infected Ghanaian adults are females (UNAIDS, 2020), while data on gut microbiome in HIV-1 infected individuals has been accumulated mostly by analyzing cohorts including larger number of men having sex with men (MSM) in developed countries (Lozupone et al., 2013; McHardy et al., 2013; Vujkovic-Cvijin et al., 2013; Dillon et al., 2014; Mutlu et al., 2014; Vujkovic-Cvijin and Somsouk, 2019). In the present study, we compared fecal microbiome in HIV-1 infected and uninfected groups matched on gender, age, and community of residence in Ghana. Analysis revealed the characteristics of dysbiotic fecal microbiome in HIV-1 infected Ghanaians.

## Materials and Methods

### Study Population

In a cross-sectional case-control study, we enrolled matched pairs of HIV-1 infected (HIV+) and uninfected (seronegative; HIV-) individuals. HIV-1 infected individuals routinely attending an HIV/AIDS clinic at the Eastern Regional Hospital, Koforidua (RHK), Ghana, were enrolled into the study. HIV-1 infected participants were identified to reside in 7 communities (Tafo, Suhum, Koforidua, Nkurakan, Jumapo, Oyoko, and Akwadum) in the Eastern Region of Ghana (**Supplementary Information 1**). HIV-1 seronegative individuals were recruited during a community health screening in these communities. Seronegative individuals matched by age (± 2 years), gender and community of residence were enrolled as controls. Only adults above 18 years old were enrolled in this study. Seronegative participants who took antibiotics within 4 weeks prior to sample collection were not enrolled. Data on HIV- was described previously (Parbie et al., 2020).

### Sample Collection

Venous blood and stool samples were collected from enrolled participants. All biological samples were transported to Noguchi Memorial Institute for Medical Research (NMIMR), University of Ghana and processed for storage within 24 hours of sample collection. Plasma and peripheral blood mononuclear cells (PBMC) were prepared from venous blood. Stool samples were collected and stored at -80°C until DNA extraction.

### Bacteria Fraction Preparation From Fecal Samples

Bacterial pellets were prepared from frozen fecal samples as previously described (Morita et al., 2007; Parbie et al., 2020) with minor modifications. Briefly, 1 g of stool was washed three times with 3 ml of SM-plus buffer (100 mM NaCl, 50 mM Tris-HCl [pH7.4], 8 mM MgSO4-7H_2_O, 5 mM CaCl2-2H_2_O, 0.01% [w/v] Gelatin) and centrifuged at 6,000 x *g* for 5 min. Then, pellets were resuspended in 20 ml of SM-plus buffer and filtered through a 100-μm cell strainer (Corning, Tokyo, Japan). One ml out of the filtrated 20 ml of bacterial suspension was used for DNA extraction.

### DNA Extraction, Amplification, and 16S rRNA Gene Sequencing

DNA was extracted from fecal sample-derived bacteria fraction as previously described (Kim et al., 2013). Gene libraries for the hypervariable V3-V4 region of 16S rRNA was prepared as previously described (Parbie et al., 2020) according to the 16S Metagenomic Sequencing Library Preparation guide (Illumina, San Diego, USA; Part # 15044223 Rev. B). Sequencing was performed on the Illumina MiSeq using MiSeq Reagent Kit v3 (600-cycle) with a 20% PhiX (Illumina) spike-in at NMIMR.

### Sequence Analyses

Sequences were quality filtered, denoised and analyzed with the Quantitative Insights Into Microbial Ecology 2 (QIIME 2™ version 2019.4) (Bolyen et al., 2019) (**Supplementary Information 2**). Briefly, paired-end reads were denoised into amplicon sequence variants (ASVs) with DADA2 (Callahan et al., 2016). Taxonomy was assigned to the resulting ASVs against the SILVA database (release 132) (Quast et al., 2012), trimmed to the V3-V4 region of the 16S rRNA gene, using Naive Bayesian classifier (Fabian et al., 2011). To remove low abundant or rare taxa prior to differential abundance analysis, sequences were preprocessed based on methodology described in ANCOM-II (Kaul et al., 2017). Differentially abundant taxa by HIV-1 status were identified using the analysis of composition of microbiome (ANCOM) and linear discriminant analysis (LDA) effect size (LEfSe) methods (Segata et al., 2011; Mandal et al., 2015).

### Analysis of Plasma Markers for Microbial Translocation

Plasma lipopolysaccharide binding protein (LBP), soluble CD14 (sCD14), and intestinal fatty acid-binding protein (I-FABP) levels were measured by enzyme-linked immunosorbent assay (ELISA) according to manufacturer specifications (R&D Systems, Minneapolis, MN, USA).

### Statistical Analyses

Data were obtained from 2017 to 2019. GraphPad Prism version 7.04 and R 3.6.0 packages were used for statistical analyses. Comparison between categorical variables between groups were performed with Fisher’s exact test. Continuous variables were assessed using Wilcoxon rank-sum test, Mann-Whitney test, or Kruskal Wallis test. Multiple test correction was performed when assessing differences between groups using Benjamini, Krieger and Yekutieli FDR correction. *p* values less than 0.05 were considered significant. To test associations involving bacteria taxa showing significant difference in abundance by HIV-1 status and clinical and immunological markers, correlation analysis was performed using R package Hmisc v4.5. Multivariate analysis of phenotypes with significant correlation was performed to ascertain association using Microbiome Multivariable Associations with Linear Models, MaAsLin2 (Mallick et al., 2021).

## Results

### Cohort Clinical Characteristics

A total of 110 participants consisting of 55 HIV-1 infected (HIV+) and 55 uninfected (HIV-) adults were enrolled in the present study. The median age of all participants was 45.5 years (IQR, 34-51) and 86 (78%) were females. Fifty-two of the HIV-1 infected participants (95%) had been on ART for a median duration of 62 months (IQR, 26-105) (**Table 1**) and 32 of them were controlling viremia (< 1,000 copies/mL). The ART regimen consisted of two kinds of nucleoside reverse transcriptase inhibitors and a non-nucleoside reverse transcriptase inhibitor (either efavirenz [n = 42] or nevirapine [n = 10]). Out of the 3 ART naïve HIV-1 infected participants, 2 had undetectable plasma viral RNA (< 20 copies/mL). The median duration since last co-trimoxazole treatment was 72 months (IQR, 24-108) in the HIV-1 infected individuals.

**Table 1:** Clinical and demographic characteristics.

Description	Seronegative (n = 55)	HIV-1 infected (n = 55)	p-value^1^
Age (yrs): median (IQR^2^)	45 (33-51)	46 (35-51)	0.741
Gender: female number (%)	43 (78%)	43 (78%)	> 0.99
CD4 cell count (cells/μl): median (IQR)	ND^3^	375 (213 - 592)	ND^3^
CD8 cell count (cells/μl): median (IQR)	ND^3^	1,345 (988 - 1,670)	ND^3^
ART duration (months): median (IQR) (n = 52)	ND^3^	62 (26 - 105)	ND^3^
Viral load (copies/ml): median (IQR) (n = 36)	ND^3^	2.7 x 10^3^ (3.5 x 10^2^ - 4.7 x 10^4^)	ND^3^
Duration since Co-trimoxazole usage (months): median (IQR)	ND^3^	72 (24 - 108)	ND^3^
sCD14 (μg/ml): mean ± SD	0.513 ± 0.449	0.906 ± 0.993	0.0427
IFABP (μg/ml): mean ± SD	0.001 ± 0.001	0.002 ± 0.002	0.0140
LBP (μg/ml): mean ± SD	4.873 ± 2.965	4.840 ± 1.619	0.6613

^1^Statistical comparison between seronegative controls and HIV-1 infected individuals was performed by Wilcoxon rank sum test or Fisher’s exact test (gender).

^2^interquartile range.

^3^not determined.

### Fecal Microbiome Diversity in HIV-1 Infected and Uninfected Adults in Ghana

**Figure 1:**
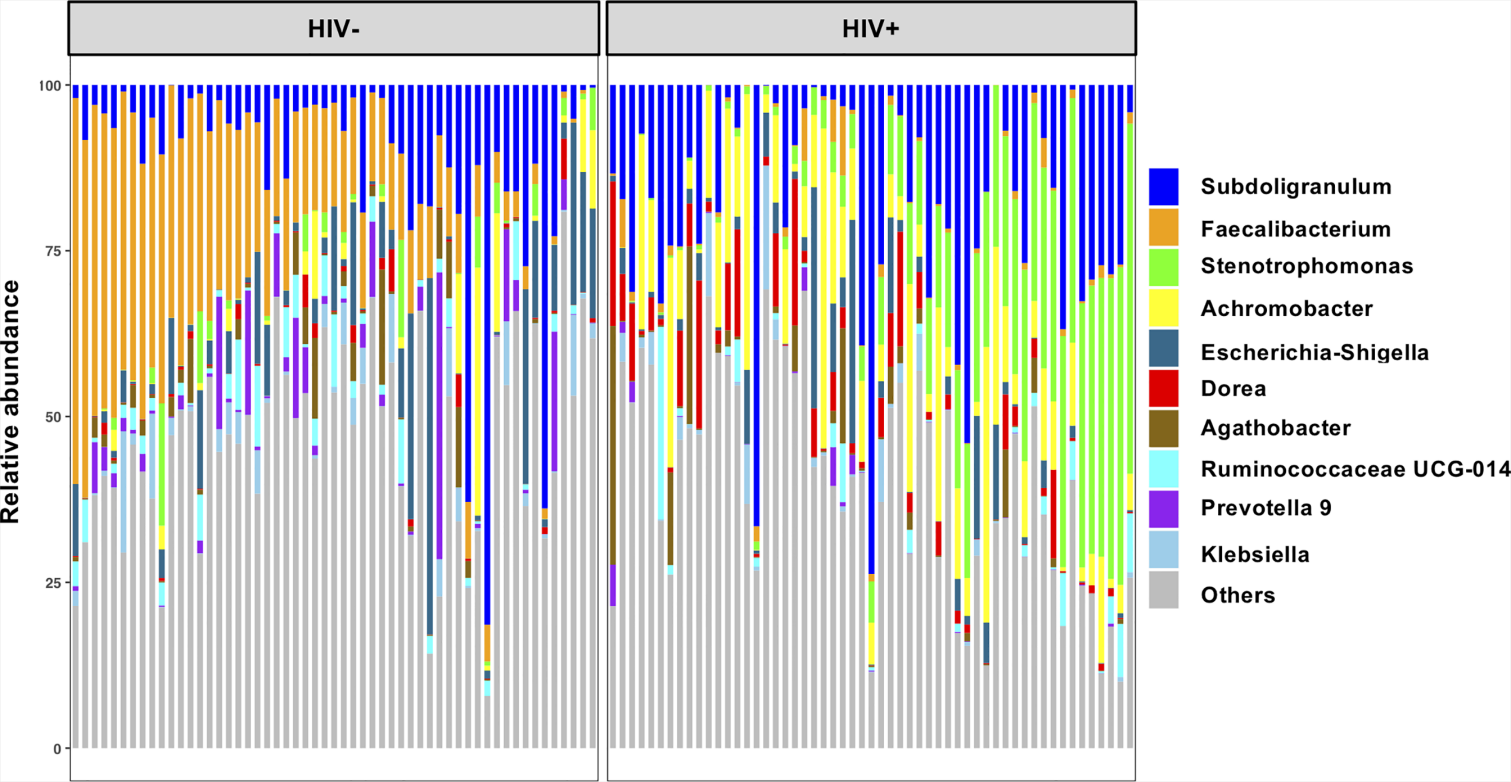
Top 10 abundant genera in fecal microbiome in all participants in the present study. Taxa bar plots showing the top 10 abundant genera in all participants (n = 110) are shown. Each bar represents frequencies of the genera in fecal microbiome of an individual. HIV-, HIV-1 uninfected individuals (n = 55); HIV+, HIV-1 infected individuals (n = 55).

The 16S rRNA gene libraries were prepared from fecal sample-derived bacteria fraction and subjected to next generation sequencing. Fecal microbiome compositions in the healthy seronegative Ghanaian adults were described previously (Parbie et al., 2020). Mean relative abundance showed that *Firmicutes* is the most abundant at the phylum level in the entire study cohort (**Supplementary Figure 1**). *Proteobacteria*, *Bacteroidetes*, and *Actinobacteria* were also abundant in HIV-, while more abundant *Proteobacteria* but less *Bacteroidetes* were observed in HIV+ individuals. At the genus level, *Faecalibacterium* and *Subdoligranulum* belonging to *Firmicutes* were dominant in HIV-, whereas *Faecalibacterium* was depleted and *Stenotrophomonas* and *Achromobacter* belonging to *Proteobacteria* as well as *Subdoligranulum* looked abundant in HIV+ (**Figure 1**).

**Figure 2:**
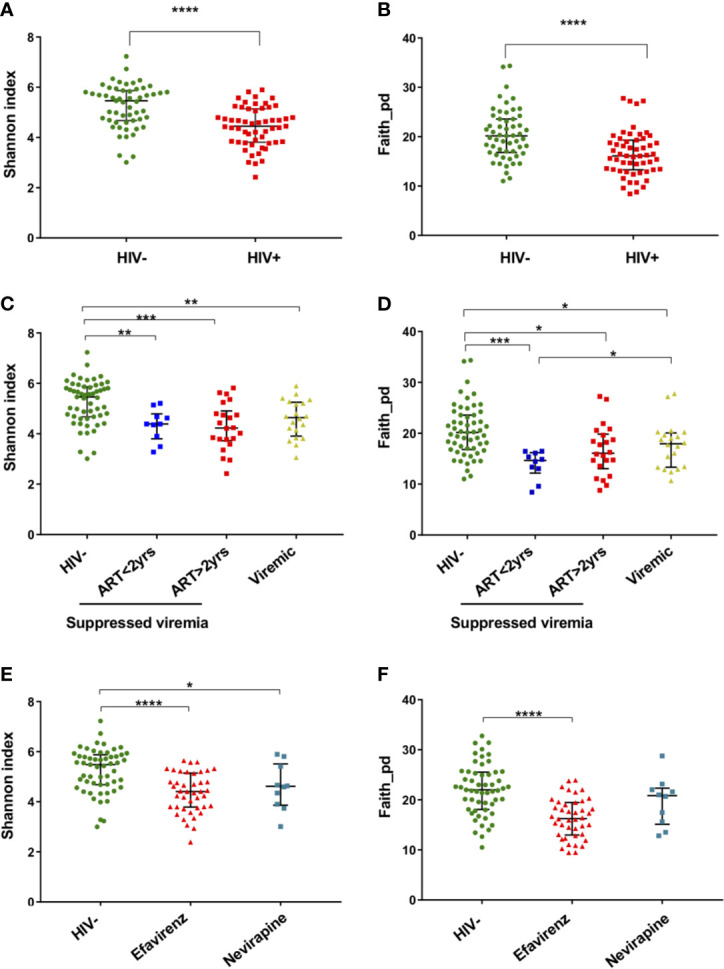
Alpha diversity of fecal microbiome in HIV-1 uninfected and infected Ghanaian adults. **(A)** Comparison of Shannon diversities of fecal microbiome between HIV- (n = 55) and HIV+ (n = 55). HIV- showed significantly higher diversity than HIV+ (*p* < 0.001). **(B)** Comparison of Faith phylogenetic diversities between HIV- and HIV+. HIV- showed significantly higher diversity than HIV+ (*p* < 0.001). **(C)** Comparison of Shannon diversities among HIV-, HIV-1 infected controllers (viral load < 1,000 copies/ml) under ART for less than 2 years (ART<2 yrs, n = 10), HIV-1 infected controllers under ART for more than 2 years (ART>2 yrs, n = 22), and HIV-1 infected non-controllers (viral load > 1,000 copies/ml) under ART (Viremic, n = 20). HIV- showed significantly higher diversity than ART<2yrs (*p* < 0.01), ART>2yrs (*p* < 0.005), and Viremic (*p* < 0.01), respectively. **(D)** Comparison of Faith phylogenetic diversities among HIV-, ART<2 yrs, ART>2 yrs, and Viremic. HIV- showed significantly higher diversity than ART<2yrs (*p* < 0.005), ART>2yrs (*p* < 0.05), and Viremic (*p* < 0.05), respectively. ART<2yrs showed significantly lower diversity than Viremic (*p* < 0.05). **(E)** Comparison of Shannon diversities among HIV-, HIV+ on ART with Efavirenz (n = 42), and HIV+ on ART with Nevirapine (n = 10). **(F)** Comparison of Faith phylogenetic diversities among HIV-, HIV+ with Efavirenz, and HIV+ with Nevirapine. Significant difference was determined by Wilcoxon rank sum test **(A, B)** or Kruskal Wallis test with Benjamini, Krieger and Yekutieli FDR correction **(C–F)**; *, **, ***, and **** indicate significant differences with *p* < 0.05, *p* < 0.01, *p* < 0.005, and *p* < 0.001, respectively.

Analyses of Chao’s richness and Fisher’s richness (Fisher et al., 1943; Chao, 1984) showed significantly lower richness in fecal microbiome of HIV+ than HIV- (**Supplementary Figures 2A, B**). Analyses of Shannon’s index and Faith’s phylogenic diversity (Shannon and Weaver, 1949; Faith, 1992) indicated significant reduction in alpha diversity in HIV+ compared to HIV- (**Figures 2A, B**). Comparison in alpha diversity between groups with low (< 200) and high (> 200) CD4 counts, short (< 2 years) and long (> 2 years) period of ART, or low (< 1,000 copies/ml) and high (> 1,000 copies/ml) plasma viral loads showed no significant difference in HIV-1 infected individuals (data not shown). However, a tendency of higher microbial richness in ART-treated HIV-1 non-controllers with > 1,000 copies/ml of plasma viral load than ART-treated HIV-1 controllers was observed (**Figures 2C, D** and **Supplementary Figures 2C, D**). While it has been indicated that different ART regimens may have different impacts on gut microbiome (Pinto-Cardoso et al., 2017; Ray et al., 2020; Imahashi et al., 2021), no significant difference in fecal microbiome richness or diversity was observed between individuals on ART with efavirenz and nevirapine in our cohort (**Figures 2E, F** and **Supplementary Figures 2E, F**).

**Figure 3:**
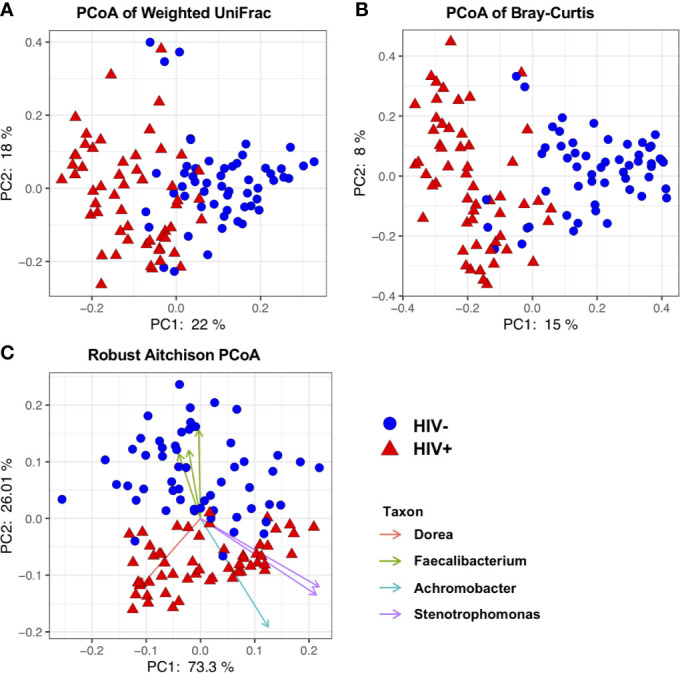
Beta diversity of fecal microbiome in HIV-1 uninfected and infected Ghanaian adults. **(A)** Principal Coordinates Analysis (PCoA) of weighted Unifrac distances in fecal microbiome in HIV- and HIV+ individuals. **(B)** PCoA of Bray-Curtis distances in HIV- and HIV+. **(C)** PCoA of Aitchison distances in HIV- and HIV+. Seven representative amplicon sequence variants (ASV) influencing the observed segregation are shown. These analyses indicate segregation between HIV- and HIV+.

Beta-diversity (similarity/dissimilarity) in fecal microbiome was assessed by weighted Unifrac, Bray-Curtis, and robust Aitchison metrics (Sorensen, 1948; Lozupone et al., 2007; Martino et al., 2019). Principal coordinates analyses (PCoA) using these matrices revealed segregation of fecal microbiome compositions between HIV- and HIV+ (**Figure 3**). This was confirmed by ADONIS permutation-based statistical test (Anderson, 2001) showing significant differences based on HIV status with *r* ^2^ of approximately 0.32, 0.11, and 0.16 in Aitchison, Bray-Curtis, and Weighted Unifrac PCoA, respectively (**Table 2**). The impact of difference in gender, age, CD4 counts or viral loads on beta-diversity in fecal microbiome was not clear (**Table 2**).

**Table 2:** Summary of ADONIS permutation-based statistical test on beta diversity metrices.

Beta Diversity Distances	Metadata Category	*r* ^2^	*p* values
Aitchison	Sex	0.000236205	0.980
	Age	0.006285174	0.386
	Community of residence	0.069178520	*0.024
	**HIV status**	**0.323352850**	*0.001
	CD4 count (below or above 250 cells/μl)	0.023073580	*0.039
	Viral load (below or above 1000 copies/ml)	0.006648337	0.355
Bray Curtis	Sex	0.008710964	0.364
	Age	0.007856389	0.499
	Community of residence	0.043018379	0.371
	**HIV status**	**0.110867018**	*0.001
	CD4 count (below or above 250 cells/μl)	0.011006354	0.119
	Viral load (below or above 1000 copies/ml)	0.012170838	0.080
Weighted Unifrac	Sex	0.014014387	0.090
	Age	0.005734581	0.635
	Community of residence	0.039710769	0.427
	**HIV status**	**0.157668889**	*0.001
	CD4 count (below or above 250 cells/μl)	0.016253230	0.050
	Viral load (below or above 1000 copies/ml)	0.019034098	*0.016

*Asterisks indicate significant difference. Metadata categories with significant p-value and r^2^ > 0.1 are shown in bold fonts.

### Fecal Microbiome Composition in HIV-1 Infected and Uninfected Adults in Ghana

**Figure 4:**
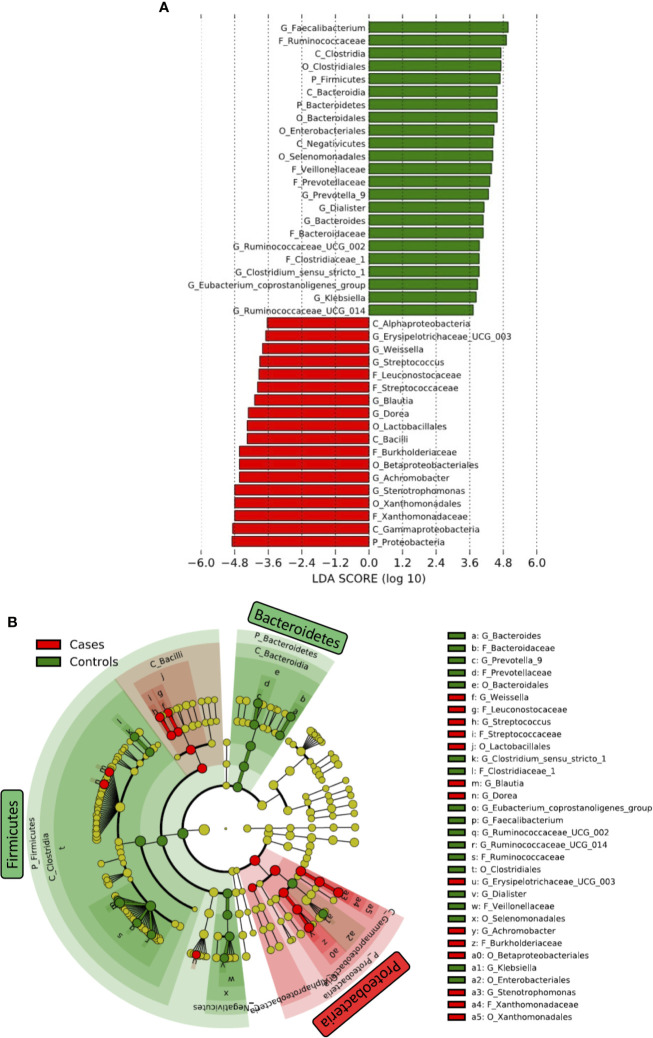
Difference in abundant taxa in fecal microbiome between HIV-1 uninfected and infected Ghanaian adults. **(A)** Data determined by linear discriminant analysis (LDA) effect size (LEfSe). Alpha value of 0.01 and 3.6 threshold on the logarithmic LDA score for discriminative features was used. P_, C_, O_, F_, G_ represent Phylum, Class, Order, Family, and Genus, respectively. **(B)** Cladogram. Green and Red indicate HIV- (Controls) and HIV+ (Cases), respectively.

Differences in taxa in fecal microbiome between HIV- and HIV+ were first determined by linear discriminant analysis (LDA) effect size (LEfSe) (Segata et al., 2011) (**Figure 4**). *Firmicutes* and *Bacteroidetes* were significantly more abundant in HIV- compared to HIV+. The abundance of *Firmicutes* was driven by the families of *Ruminococcaceae*, *Veillonellaceae*, and *Clostridiaceae_1*, while the abundance of *Bacteroidetes* was driven by the families of *Prevotellaceae* and *Bacteroidaceae* in HIV-. In contrast, *﻿Proteobacteria* was significantly more abundant in fecal microbiome of HIV+ compared to HIV-. The abundance of *Proteobacteria* in HIV+ was driven by the families of *Xanthomonadaceae* and *Burkholderiaceae*. In addition, *Streptococcaceae* belonging to *Firmicutes* was abundant in HIV+.

We also determined several significant discriminating taxa at the genus level by LEfSe and analysis of composition of microbiome (ANCOM) (Mandal et al., 2015; Kaul et al., 2017) comparing HIV- and HIV+ (**Figure 4** and **Supplementary Figure 3**). *Faecalibacterium, [Eubacterium] coprostanoligenes group, Ruminococcaceae UCG-002, Bacteroides, Prevotella_9, Butyricimonas, Dialister, Phascolarctobacterium, Ruminococcaceae UCG-014, Parabacteroides, Ruminococcaceae UCG-005, Clostridium sensu_stricto_1*, and *Klebsiella* were more abundant in HIV-. In contrast, *Stenotrophomonas, Dorea, Achromobacter, Blautia, Streptococcus, Subdoligranulum, Erysipelotrichaceae UCG-003, Ochrobactrum, Weissella*, and *Gemella* were more abundant in HIV+.

### Association of Fecal Microbiome Diversity With Clinical Markers

**Figure 5:**
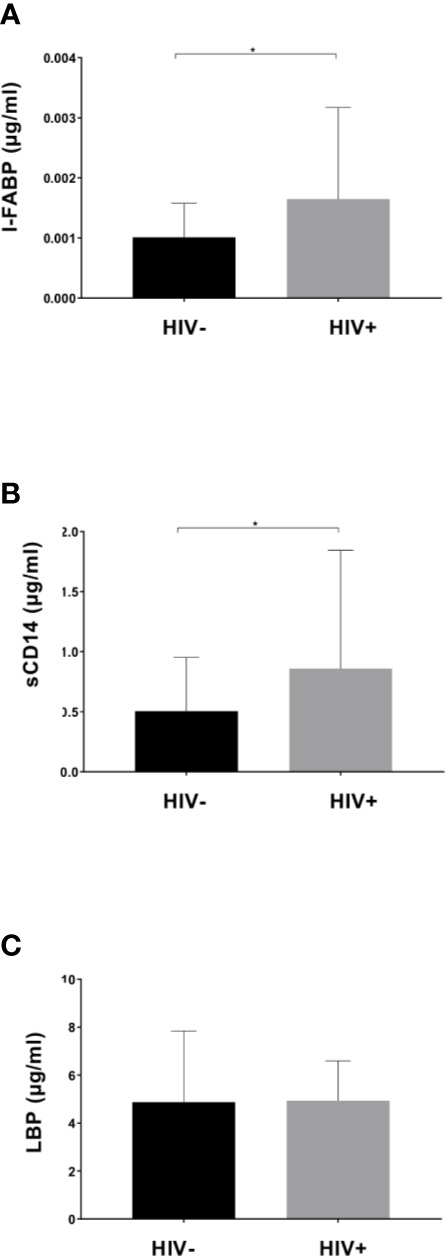
Comparison of plasma markers between HIV-1 uninfected and infected Ghanaian adults. Comparisons of plasma I-FABP **(A),** sCD14 **(B)**, and LBP **(C)** levels are shown. HIV+ (n = 55) showed significantly higher I-FABP and sCD14 levels than uninfected HIV- (n = 55) (*p* < 0.05 [*]; Wilcoxon rank sum test).

**Figure 6:**
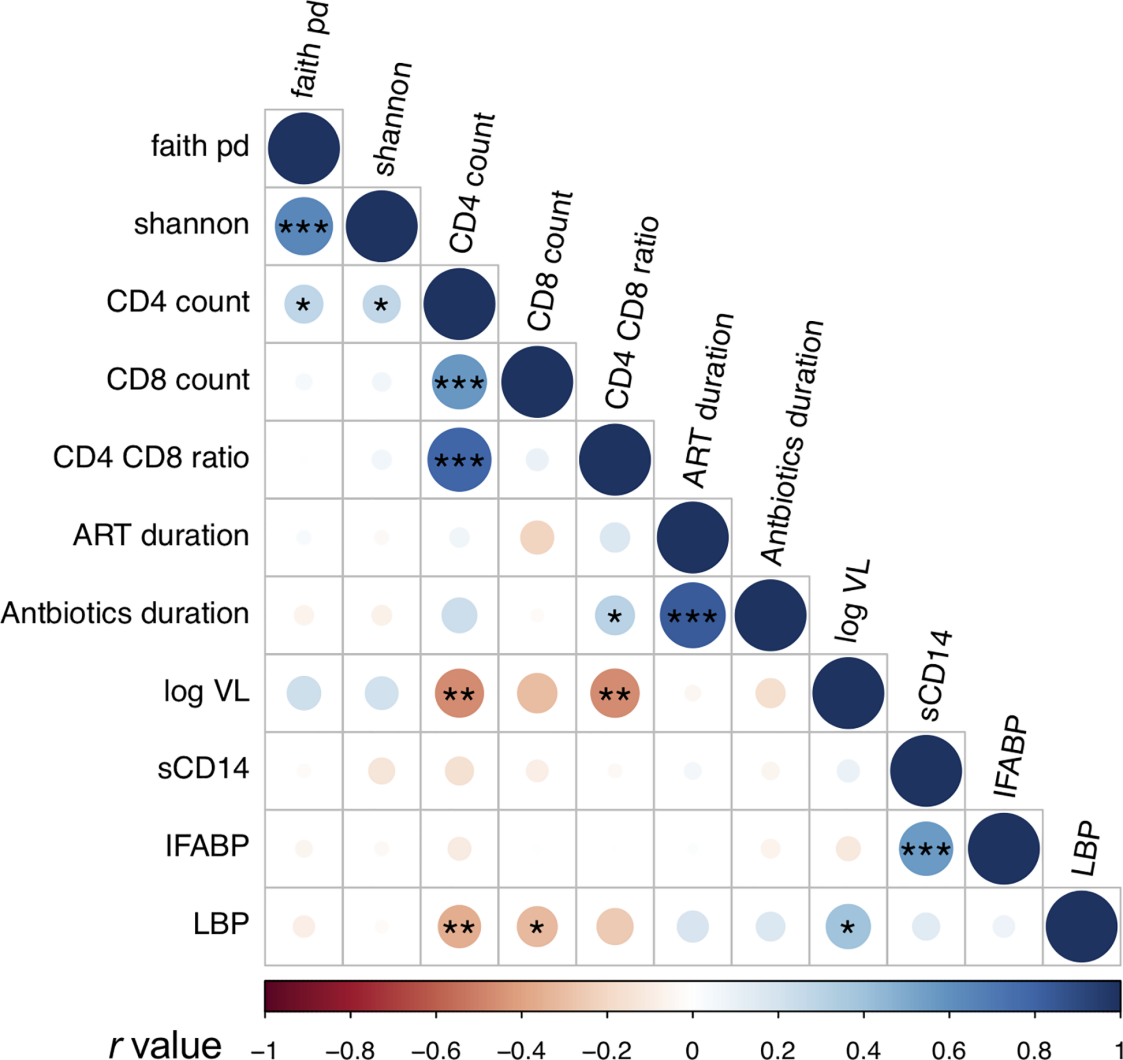
Analyses of correlation between fecal microbiome diversity and clinical markers. Asterisks indicate significant correlation determined by Spearman’s test; *, **, and *** indicate significant differences with *p* < 0.05, *p* < 0.01 and *p* < 0.005, respectively. Circle sizes are proportional to strength of correlation. Faith_pd, Faith’s phylogenetic diversity; shannon, Shannon’s index; ART_duration, duration under ART; Antibiotics_duration, duration since last Co-trimoxazole treatment; log_VL, viral load; sCD14, plasma soluble CD14 levels; IFABP, plasma intestinal fatty acid-binding protein levels; LBP, plasma lipopolysaccharide binding protein levels. n = 110 in faith_pd, shannon, sCD14, IFABP, and LBP; n = 55 (HIV+) in CD4 count, CD8 count, and CD4_CD8_ratio; n = 52 in ART_duration; n = 54 in Antibiotics_duration, n = 36 in log_VL (> 20 copies/ml).

Plasma levels of I-FABP, sCD14, and LBP, markers for microbial translocation (Younas et al., 2019), were also examined. Analysis revealed significantly higher levels of I-FABP and sCD14 in HIV+ than in HIV- (**Figure 5**). However, there was no significant difference in plasma LBP levels between HIV- and HIV+. Correlation analysis found a strong positive correlation between plasma I-FABP and sCD14 levels (*p* < 0.0001, *r* = 0.55) (**Figure 6**). Plasma LBP levels did not show significant correlation with I-FABP or sCD14 but were correlated negatively with CD4 counts (*p* < 0.005, *r* = -0.38) and CD8 counts (*p* < 0.05, *r* = -0.32) and positively with plasma viral load (*p* < 0.05, *r* = 0.39) (**Figure 6** and **Supplementary Figure 4**). These microbial translocation markers exhibited no significant correlation with alpha diversity of fecal microbiome (**Figure 6**). However, the alpha diversity showed a moderate but significant positive correlation with CD4 count (Faith_pd: *p* < 0.05, *r* = 0.28; Shannon: *p* < 0.05, *r* = 0.27) (**Figure 6** and **Supplementary Figure 4**).

**Figure 7:**
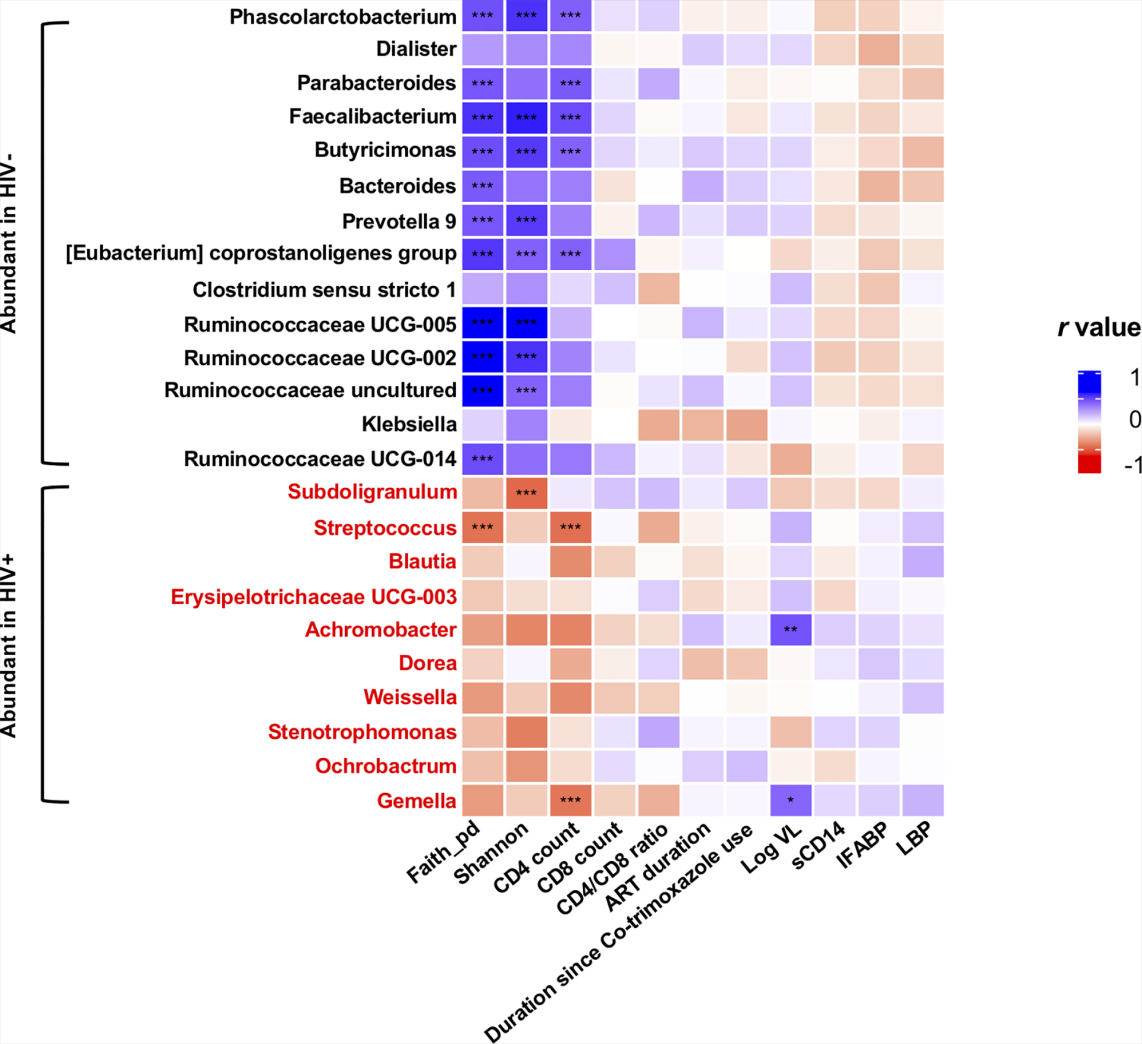
Analyses of correlation of genera showing significant difference in abundance in HIV- and HIV+ with clinical and immunological markers and microbiome diversities. Faith_pd, Faith’s phylogenetic diversity (n = 110); shannon, Shannon’s index (n = 110); CD4 count (n = 55); CD8 count (n = 55); CD4_CD8_ratio (n = 55); ART_duration, duration under ART (n = 52); Duration since Co-trimoxazole use (n = 54); Log VL, viral load (> 20 copies/ml) (n = 36); sCD14, plasma soluble CD14 levels (n = 110); IFABP, plasma intestinal fatty acid-binding protein levels (n = 110); LBP, plasma lipopolysaccharide binding protein levels (n = 110). Asterisks indicate significant correlation determined by Spearman’s test; *, **, and *** indicate significant differences with *p* < 0.05, *p* < 0.01, and *p* < 0.005, respectively. Significantly abundant genera were determined by ANCOM and LEfSE. Genera significantly abundant in HIV+ are shown by red.

In all the genera identified to be more abundant in HIV+, the relative abundance showed a trend of negative correlation with fecal microbiome alpha diversity and CD4 counts (**Figure 7**). In particular, strong inverse correlation was observed between relative abundance in *Streptococcus* and *Gemella* and CD4 counts (*p* < 0.001, *r* < -0.4) (**Supplementary Figures 5** and **9**). In contrast, the relative abundance in the genera identified to be more abundant in HIV- showed a trend of positive correlation with fecal microbiome alpha diversity and CD4 counts (**Figure 7**). In particular, relative abundance in *Phascolarctobacterium*, *Faecalibacterium*, *Butyricimonas*, and *[Eubacterium] coprostanoligenes group* showed strong positive correlation with fecal microbiome alpha diversity and CD4 counts (*p* < 0.001, *r* > 0.4) (**Supplementary Figures 6–9**).

## Discussion

Gut microbiota are known to be influenced by factors such as dietary behavior linked with socio-cultural and socio-economic status, which are different among populations (O’Keefe et al., 2007; Yatsunenko et al., 2012; Senghor et al., 2018; Hansen et al., 2019). It is thus important to obtain data on microbiome in individual populations. In sub-Saharan Africa, studies on gut microbiome in rural populations have indicated abundance in *Prevotella* which is associated with high fiber-carbohydrate diet (Yatsunenko et al., 2012; Schnorr et al., 2014; Morton et al., 2015; Monaco et al., 2016; Nowak et al., 2017; Senghor et al., 2018; Hansen et al., 2019). In industrialized populations, however, abundance in *Bacteroides* associated with high animal fat and protein has been indicated (Schnorr et al., 2014; Brewster et al., 2019). Individuals from suburban communities show abundance in *Prevotella* and *Bacteroides*, exhibiting both rural and urban features (Brewster et al., 2019). Our cohort consisting of individuals from peri-urban communities exhibited abundance in *Prevotella* and *Bacteroides* in fecal microbiome, suggesting the pattern of dietary habit in transition from rural to industrialized area (Parbie et al., 2020). The present study describes the characteristics of fecal microbiome of HIV-1 infected Ghanaians in comparison with the healthy seronegative Ghanaian control. *Faecalibacterium* (the most abundant genus) as well as *Prevotella* and *Bacteroides* were not included in the top 10 abundant taxa in HIV+, indicating loss of critical commensals in HIV-1 infected adults in our cohort.

Consistent with previous reports (Monaco et al., 2016; Nowak et al., 2017; Flygel et al., 2020), our analyses showed significant reduction in alpha diversity in HIV+ compared to HIV- (**Figure 2**). There was also a significant difference in beta diversity of fecal microbiome between HIV- and HIV+ (**Figure 3** and **Table 2**). The difference indicated by weighted Unifrac distances (which incorporates phylogeny) was considered to be mainly attributed by enrichment of *Proteobacteria* and loss of *Firmicutes* and *Bacteroidetes* in HIV+ (**Figure 4**).

Several reports have indicated that microbiome diversity decreases after ART initiation but is recovered by prolonged ART treatment (Klase et al., 2015; Nowak et al., 2017; Flygel et al., 2020). This can explain our observation of a trend of higher alpha diversity in HIV-1 controllers under longer ART (> 2 years) compared to those under shorter ART (< 2 years) in the present study (**Figures 2C, D** and **Supplementary Figures 2C, D**). Unexpectedly, microbial richness in HIV-1 non-controllers under ART looked higher than HIV-1 controllers under ART (**Figures 2C, D**). One possible speculation is poor drug compliance in HIV-1 non-controllers.

The present study confirmed differences in fecal microbiome compositions between HIV+ and HIV- in Ghana. Our analysis showed significant increase in *Proteobacteria* but decrease in *Firmicutes* and *Bacteroidetes* at the phylum level in fecal microbiome of HIV-1 infected individuals in Ghana (**Supplementary Figure 1**), similarly with the previous observations in other countries (McHardy et al., 2013; Vujkovic-Cvijin et al., 2013; McHardy et al., 2013; Monaco et al., 2016; Vujkovic-Cvijin and Somsouk, 2019). Depletion of these bacteria groups can be associated with non-AIDS disease progression (Vujkovic-Cvijin et al., 2013). However, some bacteria belonging to *Firmicutes* such as *Streptococcus* were enriched in HIV+ (**Figure 4**). Although depletion of *Lachnospiraceae* including *Dorea* and *Blautia* in HIV-1 infected individuals have been reported in several cohorts (Mutlu et al., 2014; Vázquez-Castellanos et al., 2015; Vujkovic-Cvijin and Somsouk, 2019), Ghanaian HIV+ individuals exhibited enrichment of *Dorea* and *Blautia* (**Figure 4**). Furthermore, we found a depletion of *Prevotella* in HIV+ individuals in Ghana, whereas previous studies on gut microbiome have described enrichment of *Prevotella* in HIV-1-infected populations (Lozupone et al., 2013; Dillon et al., 2014; Mutlu et al., 2014; Vujkovic-Cvijin and Somsouk, 2019). Recently, enrichment of *Prevotella* has been attributed to sexual practice in MSM independent of HIV status (Armstrong et al., 2018; Vujkovic-Cvijin and Somsouk, 2019; Vujkovic-Cvijin et al., 2020). Since females are 78% of our cohorts, our results suggest a decrease in *Prevotella* in HIV+ non-MSM Ghanaians.

The alpha diversity of fecal microbiome exhibited a significant correlation with CD4 counts (**Figure 7** and **Supplementary Figures 4D, E**). Relative abundance in the genera identified to be more abundant in HIV+ showed a trend of negative correlation with fecal microbiome alpha diversity and CD4 counts (**Figure 7**). In contrast, the relative abundance in the genera identified to be more abundant in HIV- showed a trend of positive correlation with fecal microbiome alpha diversity and CD4 counts. Enrichment of *Streptococcus* in fecal microbiome of HIV-1 infected individuals with low CD4 counts has been reported previously (Monaco et al., 2016; Shenoy et al., 2019). The inverse correlation of relative abundance in *Gemella* and *Streptococcus* with CD4 counts in the present study (**Figure 7** and **Supplementary Figures 5C, D**) may imply association of these bacteria with immunosuppression (Shenoy et al., 2019; Ueberroth and Roxas, 2019). Furthermore, this study showed reduction of several butyrate producing bacteria including *Faecalibacterium, Butyricimonas*, and other members of *Ruminococcaceae*, known for their role in maintenance of gut homeostasis (Furusawa et al., 2013), in HIV+ as previously reported (Dillon et al., 2017). These results describing genera that exhibit increase or decrease associated with disease progression could be a fundamental data for our understanding of the pathogenesis in HIV-1 infection.

Potential confounders in gut microbiome studies include nutrition, body mass index (BMI) and antibiotic usage. Unfortunately, in our cohort, information on nutrition and BMI was unavailable, while HIV- participants who took antibiotics within 4 weeks prior to sample collection were excluded. Only one HIV+ participant had taken co-trimoxazole within 1 month prior to sample collection.

Most non-African cohorts in previous reports largely included MSM, but 86 of 110 individuals are female in our cohort (Lozupone et al., 2013; McHardy et al., 2013; Vujkovic-Cvijin et al., 2013; Dillon et al., 2014; Mutlu et al., 2014; Vujkovic-Cvijin and Somsouk, 2019). Hence, we looked for available data on a cohort including substantial number of females and chose a cohort in the Netherlands (Vujkovic-Cvijin et al., 2020) as a representative non-African cohort for comparison. We used data on 37 non-MSM males (HIV- = 20, HIV+ = 17) and 39 females (HIV- = 20, HIV+ = 19) from the Dutch cohort. Comparison of Shannon’s index of fecal microbiome in Ghana and Netherlands indicated that both HIV- and HIV+ groups in our study had significantly lower alpha diversity compared to their respective counterparts from Netherlands (**Supplementary Figure 10A**). These differences were not observed in Faith’s phylogenic diversity (**Supplementary Figure 10B**). However, reduction in diversity in HIV+ compared to HIV- was more distinct in our cohort than in the Netherlands in both Shannon’s index and Faith’s phylogenic diversity. Principal coordinates analyses of Weighted Unifrac distances revealed significant differences in beta diversity between Ghanaian and Dutch HIV- and between Ghanaian and Dutch HIV+ (**Supplementary Figure 10C**).

This is the first study, although descriptive, that analyzes the fecal microbiome in HIV-1 infected Ghanaian adults using high throughput metagenomic tools. Analysis revealed significant difference in fecal microbiome diversity and compositions between HIV-1 infected and uninfected individuals. Our results describing the feature of fecal microbiome in Ghana provide valuable data in West Africa, where data on enteric microbiome has not yet been systematically accumulated, and thus contribute to understanding of the interaction between HIV-1 and enteric microbiota in a population specific manner.

## Data Availability Statement

The data and materials supporting the finding in this study are available within the publication or can be obtained upon request to the corresponding author. The sequences obtained by NGS were deposited in the DNA Data Bank of Japan (DDBJ) (https://ddbj.nig.ac.jp/DRASearch; accession number: DRA010770).

## Ethics Statement

The studies involving human participants were reviewed and approved by The Institutional Review Board of Noguchi Memorial Institute for Medical Research (NMIMR) and the Ethical Committee of National Institute of Infectious Diseases (NIID). The patients/participants provided their written informed consent to participate in this study.

## Author Contributions

PP, TMi, KI, WA, and TMa conceived and designed the experiments. SO and WA contributed to sample collection. PP, TMi, AI, AK-T, SS, CA, DK, and EB performed the experiments. PP, TMi, AI, AK-T, LR, SU, SI, YK, WA, and TMa analyzed the data. PP, TMi, HK, KI, WA, and TMa wrote the paper. All authors contributed to the article and approved the submitted version.

## Funding

This study was supported by Japan Agency for Medical Research & Development (AMED) (grant number: JP18fk0410003, JP20fk0410011, JP20fk0108125, JP20fk0108139, JP19kk0205024, and JP20jk0210002), AMED-JICA (the Science and Technology Research Partnership for Sustainable Development [SATREPS]; JP20jm0110012), and the Ministry of Education, Culture, Sports, Science, and Technology in Japan (18H02666). The Kumamoto University International Scholarship Program generously provided travel support during this study.

## Conflict of Interest

The authors declare that the research was conducted in the absence of any commercial or financial relationships that could be construed as a potential conflict of interest.
