# Supplementary material for: Dysbiotic Fecal Microbiome in HIV-1 Infected Individuals in Ghana
Source: Front Cell Infect Microbiol. 2021 May 18;11:646467. doi: 10.3389/fcimb.2021.646467 (PMC8168436; doi:10.3389/fcimb.2021.646467)
Supplement: Supplementary file 1 [file DataSheet_1.pdf]

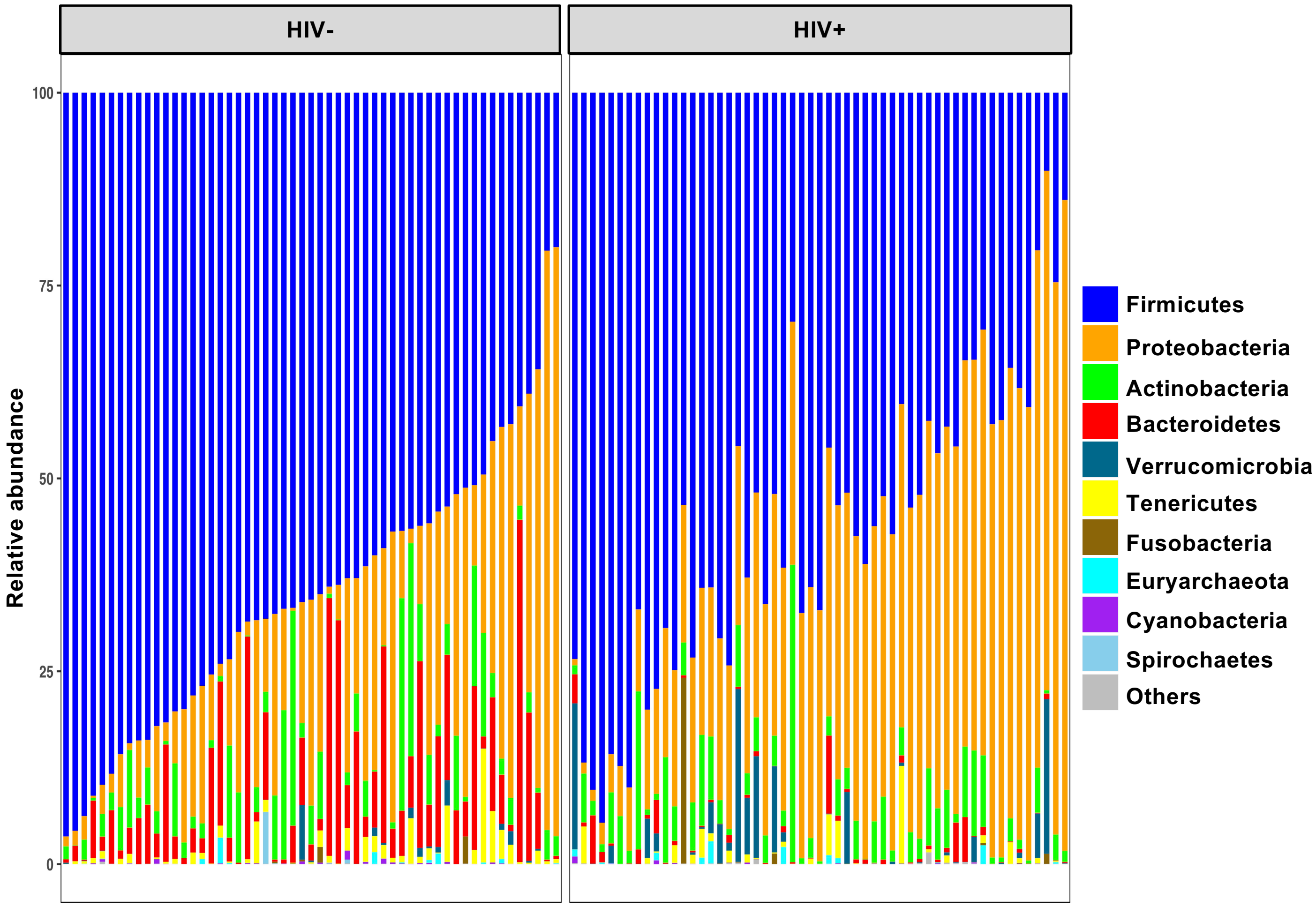

Supplementary Figure 1. Top 10 abundant phyla in fecal microbiome of all participants in the present study.

Taxa bar plots of the top 10 abundant phyla in HIV- and HIV+ are shown. Each bar represents an individual. Fecal microbiome of HIV- is dominated (mean relative abundance > 1%) at the phylum level by Firmicutes, Proteobacteria, Bacteroidetes, and Actinobacteria with mean relative abundances of 65%, 18%, 9%, and 5%, respectively.

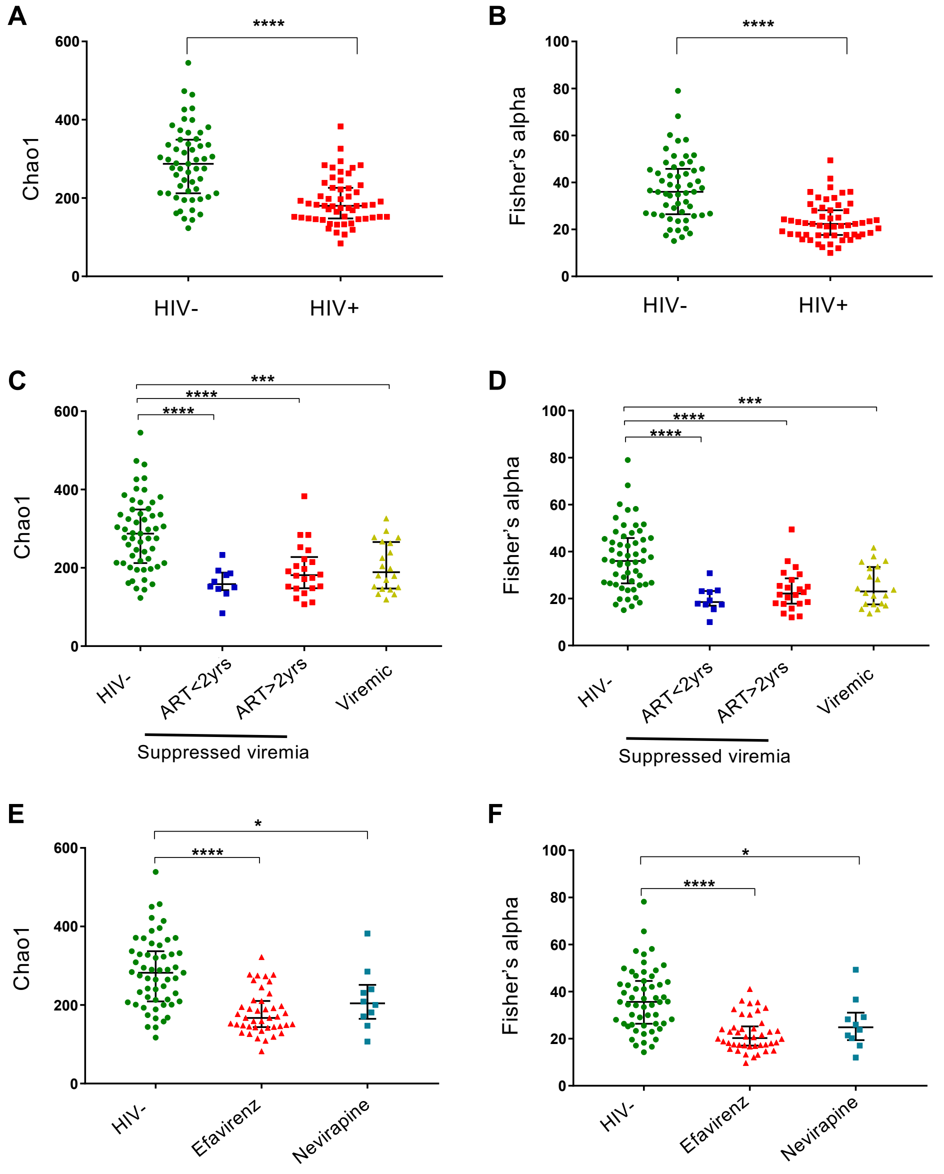

Supplementary Figure 2. Richness in fecal microbiome in HIV-1 uninfected and infected Ghanaian adults.

Comparison of Chao's richness (Chao 1) **(A, C, E)** and Fisher's richness (Fisher's alpha) **(B, D, F)** of HIV- (n = 55) with HIV+ (n = 55) **(A, B)** or HIV+ under ART (HIV-1 controllers [viral load < 1,000 copies/ml] on ART for less than 2 years [ART<2 yrs, n = 10], HIV-1 controllers on ART for more than 2 years [ART>2 yrs, n = 22], and HIV-1 non-controllers [viral load > 1,000 copies/ml] on ART [Viremic, n = 20]) **(C, D)**. **(E, F)** Compares richness among HIV+ on Efavirenz based ART (42), Nevirapine based ART (n=10) and HIV- (n=55). Significant difference was determined by Wilcoxon rank sum test (A, B) or Kruskal Wallis test with Benjamini, Krieger and Yekutieli FDR correction **(C-F)**; *** and **** indicate significant differences with $p < 0.005$ and $p < 0.001$, respectively.

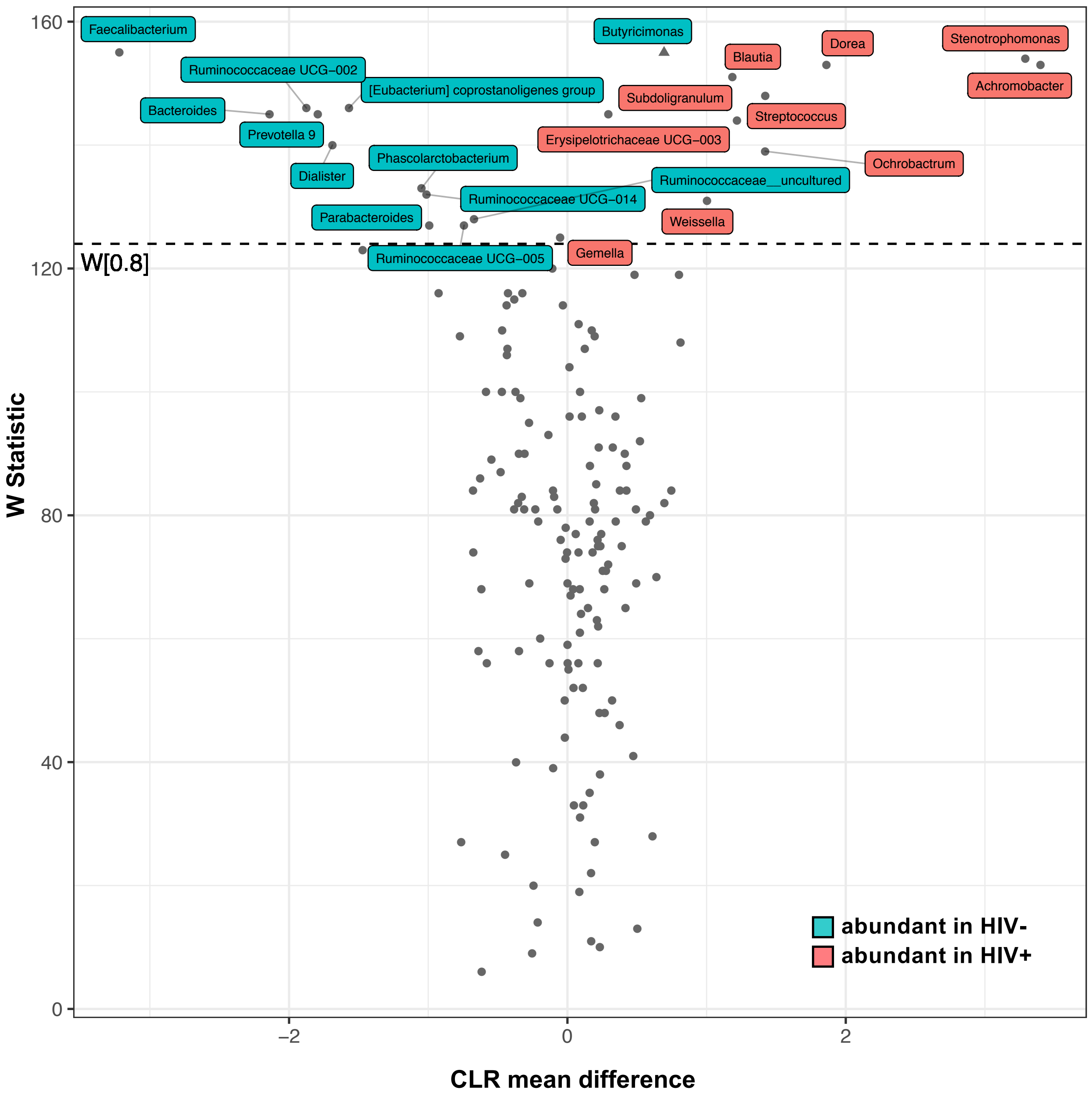

Supplementary Figure 3. Volcano plot of genera showing difference between HIV-1 uninfected and infected Ghanaian adults detected by ANCOM.
Significant difference in abundance was determined by threshold of 0.8 W-statistic and alpha < 0.05. Points represents genera. Genera showing significant difference in abundance between HIV- and HIV+ are labeled. *Butyricimonas* (indicated in triangle) was detected as structural zero (mean relative abundance in HIV+ is close to zero).

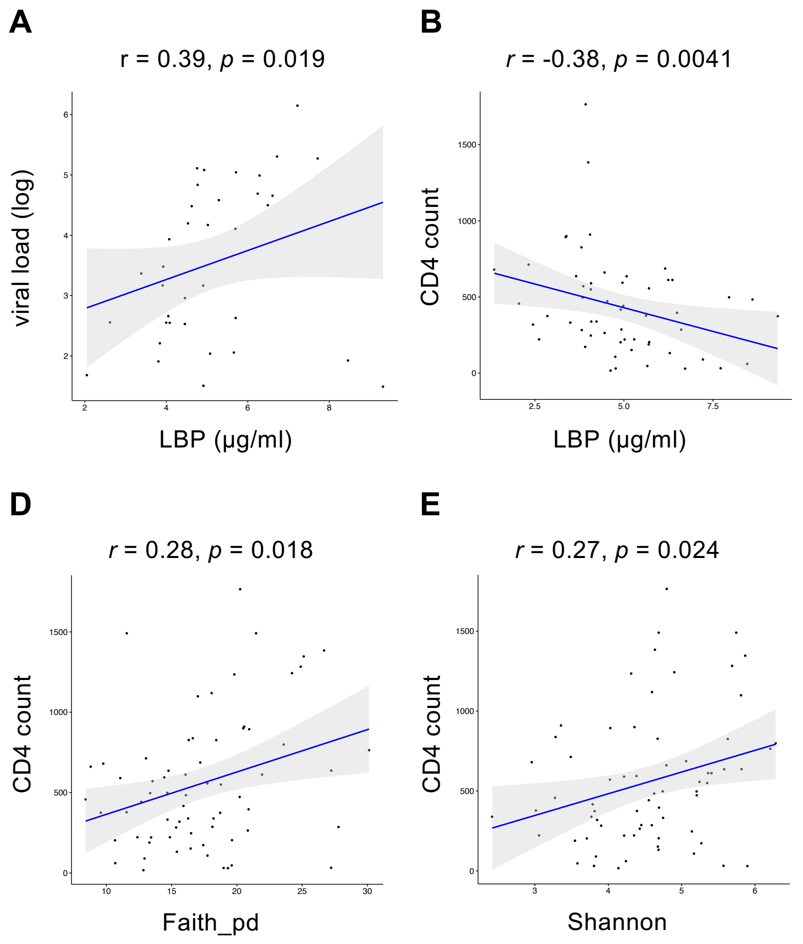

Supplementary Figure 4. Representative plots showing significant correlation indicated in Figure 6.
(A, B, and C) Significant correlation of plasma LBP levels with viral loads **(A)**, CD4 counts **(B)**, and CD8 counts **(C)** in HIV+. **(D and E)** Significant correlation of CD4 counts with alpha diversities (**D**: Faith's phylogenetic diversity [Faith_pd], **E**: Shannon's index) in fecal microbiome in HIV+.

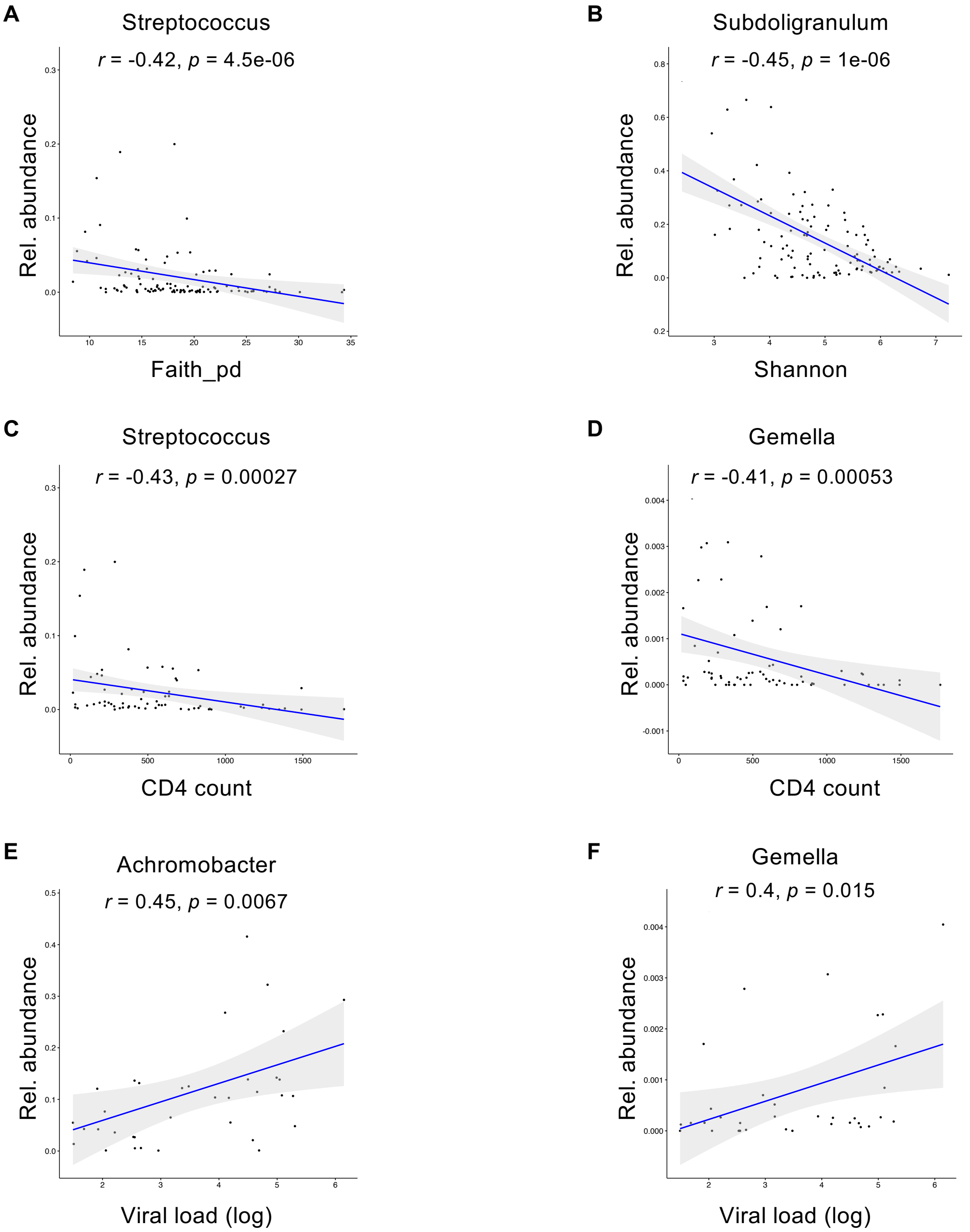

Supplementary Figure 5. Plots indicating significant correlation in genera enriched in HIV-1 infected individuals.

Plots exhibiting significant correlation with $r > |0.4|$ by Spearman's test, as indicated in Figure 7, are shown. Significant correlation of relative (rel.) abundance in the indicated genera with alpha diversities (**A**: Faith's phylogenetic diversity [Faith_pd], **B**: Shannon's index), CD4 counts **(C, D)**, and plasma viral loads **(E, F)**, respectively, are indicated. n = 110 in A and B; n = 55 in C and D (HIV+); n = 36 in E and F (HIV+ with > 20 copies/ml of viral loads).

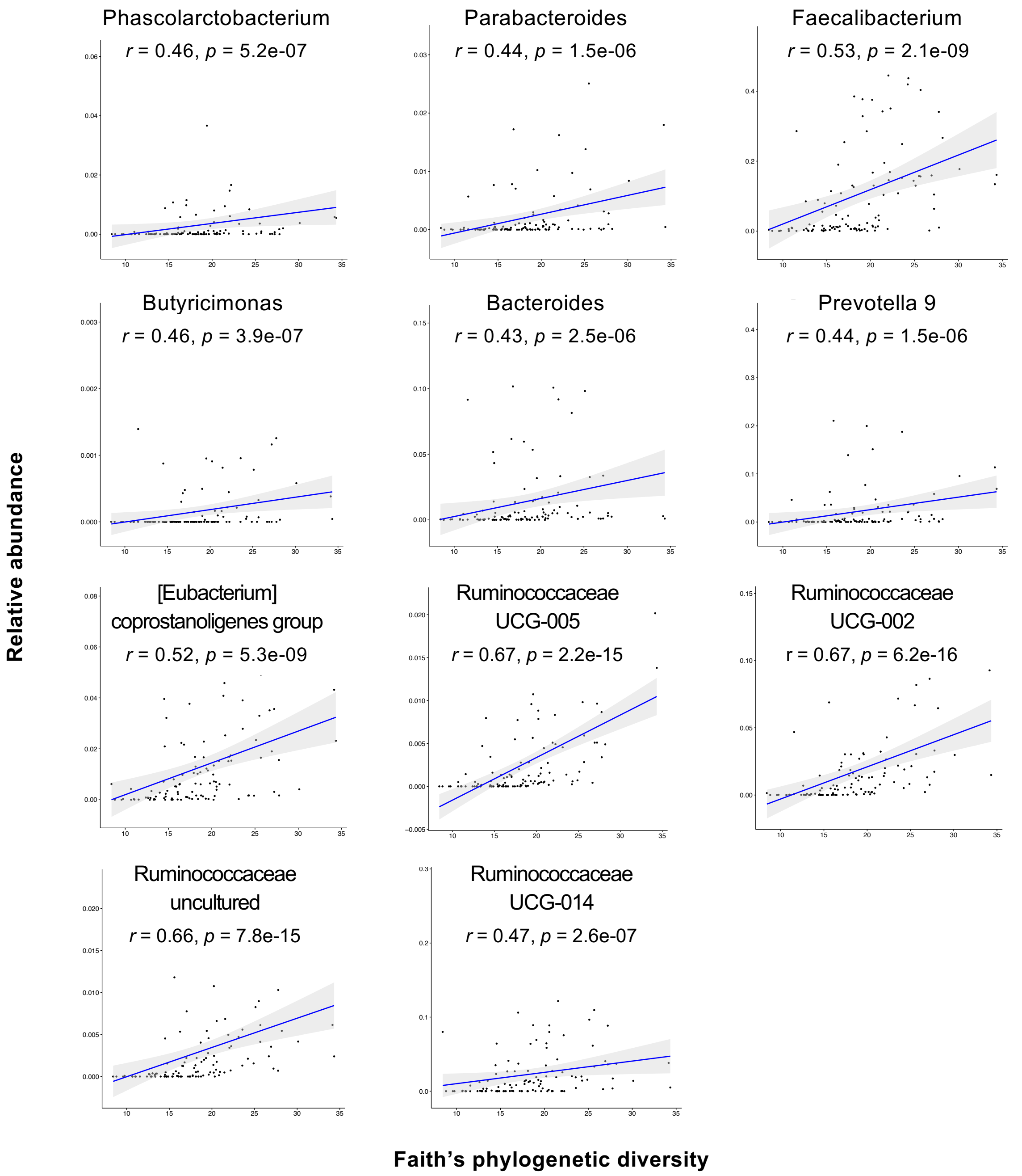

Supplementary Figure 6. Plots indicating significant correlation between Faith's phylogenetic diversity and relative abundance in genera decreased in HIV-1 infected individuals.
Plots exhibiting significant correlation with $r > |0.4|$ by Spearman's test, as indicated in Figure 7, are shown. n = 110.

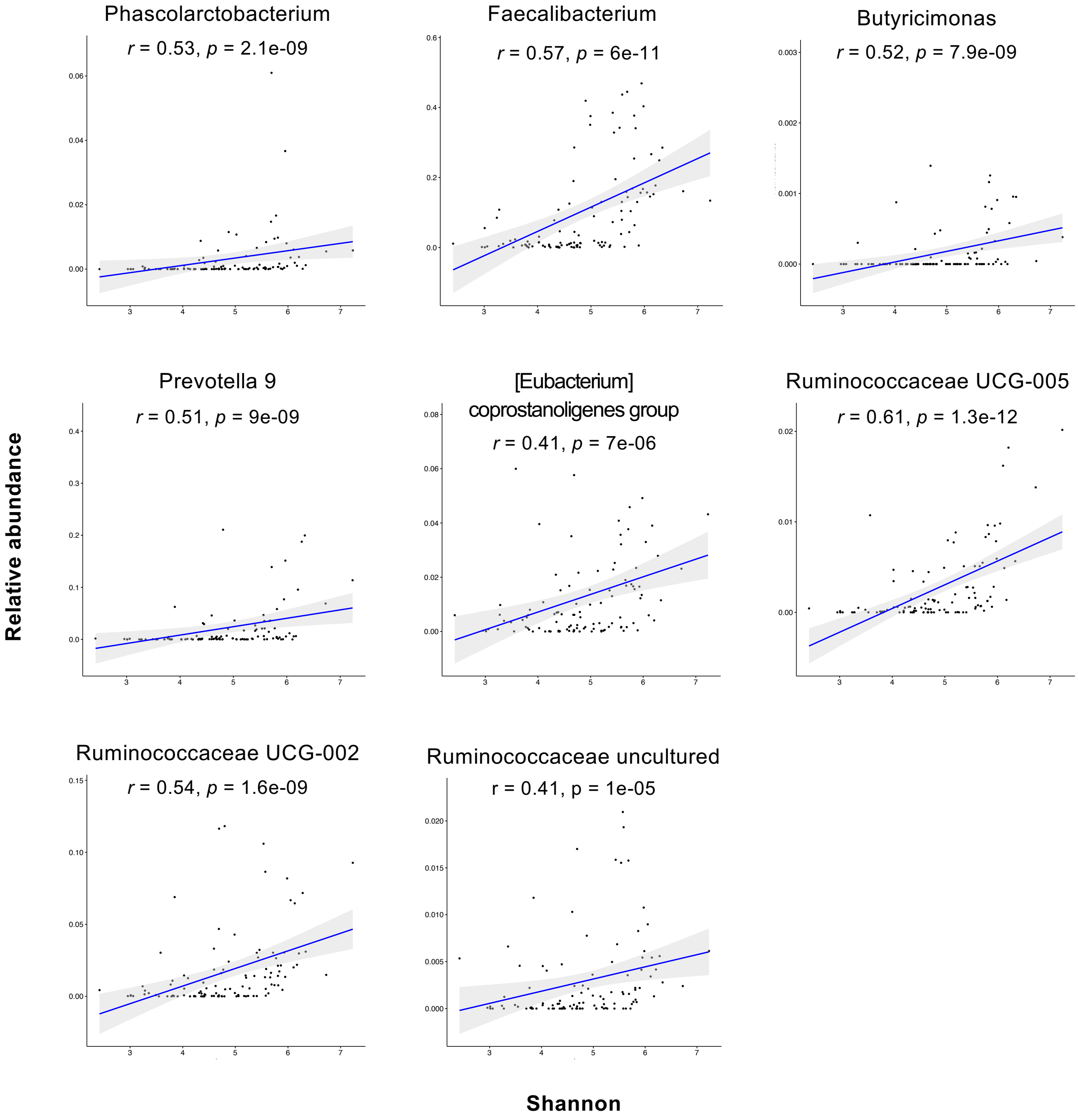

Supplementary Figure 7. Plots indicating significant correlation between Shannon's alpha diversity and relative abundance in genera decreased in HIV-1 infected individuals.
Plots exhibiting significant correlation with *r* > |0.4| by Spearman's test, as indicated in Figure 7, are shown. n = 110.

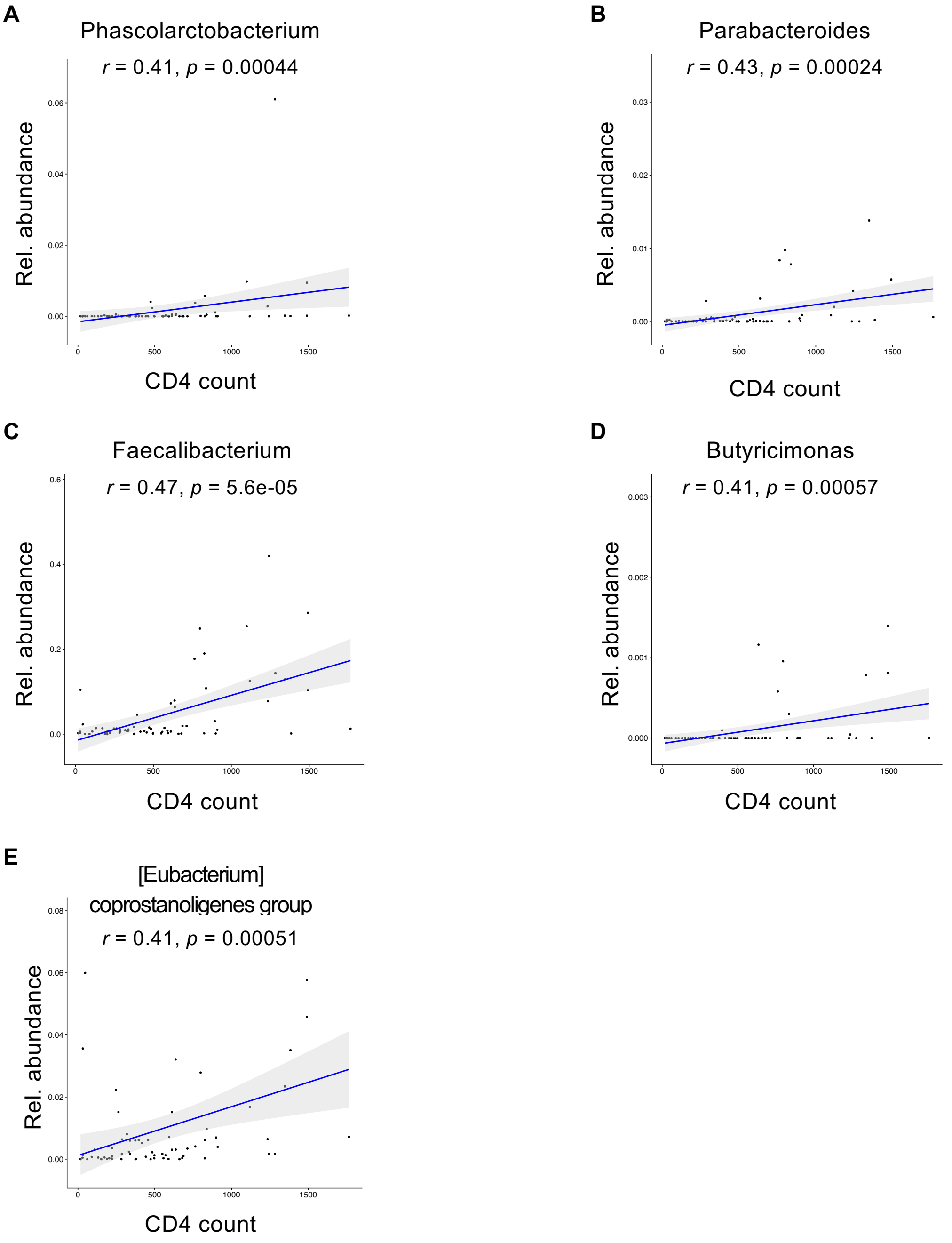

Supplementary Figure 8. Plots indicating significant correlation between CD4 counts and relative abundance in genera decreased in HIV-1 infected individuals.

Plots exhibiting significant correlation with $r > |0.4|$ by Spearman's test, as indicated in Figure 7, are shown. n = 55 (HIV+).

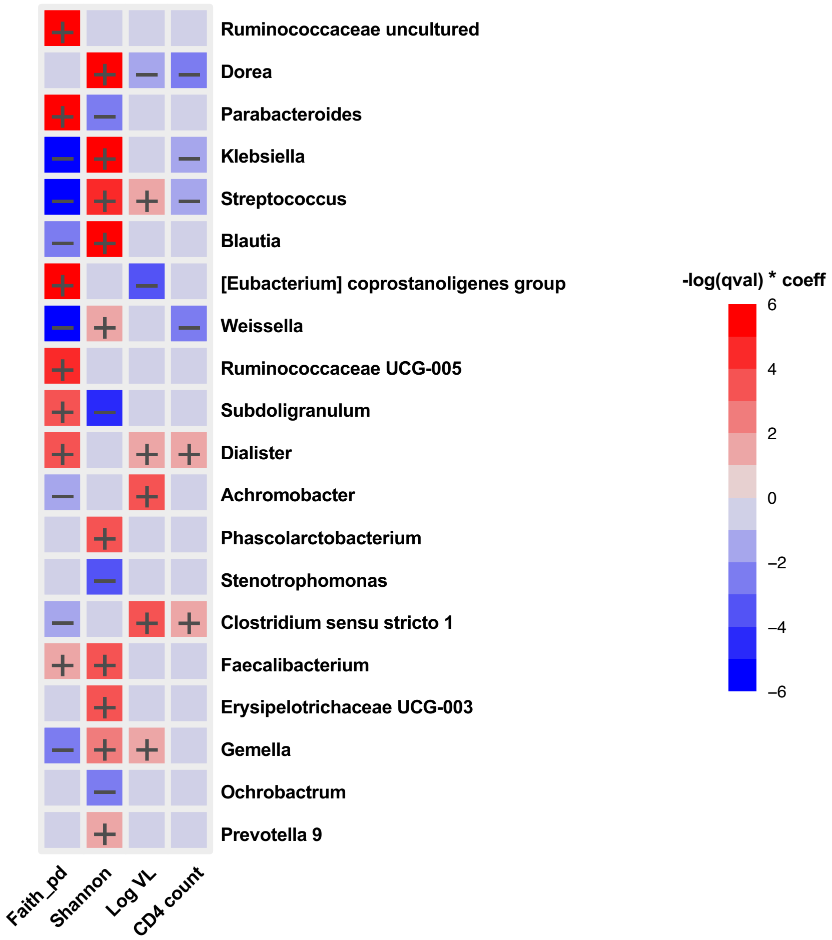

Supplementary Figure 9. Heatmap of significant associations by Microbiome Multivariable Association with Linear Models 2 (MaAsLin2).

Multivariate analysis was performed to ascertain the association of genera showing significant difference in abundance by HIV status and variables showing significant correlation (Figure 7). Genera indicating significant association are shown. The following parameters were specified in the model: minimum Prevalence= 0.4, Normalization= NONE, Transform= NONE, Analysis method= Copound Poisson Linear Model, Fixed effects = (CD4 count, log VL, faith pd, Shannon).

Coeff: Coefficient (effect size), qval: Benjamini-Hochberg adjusted p-value.

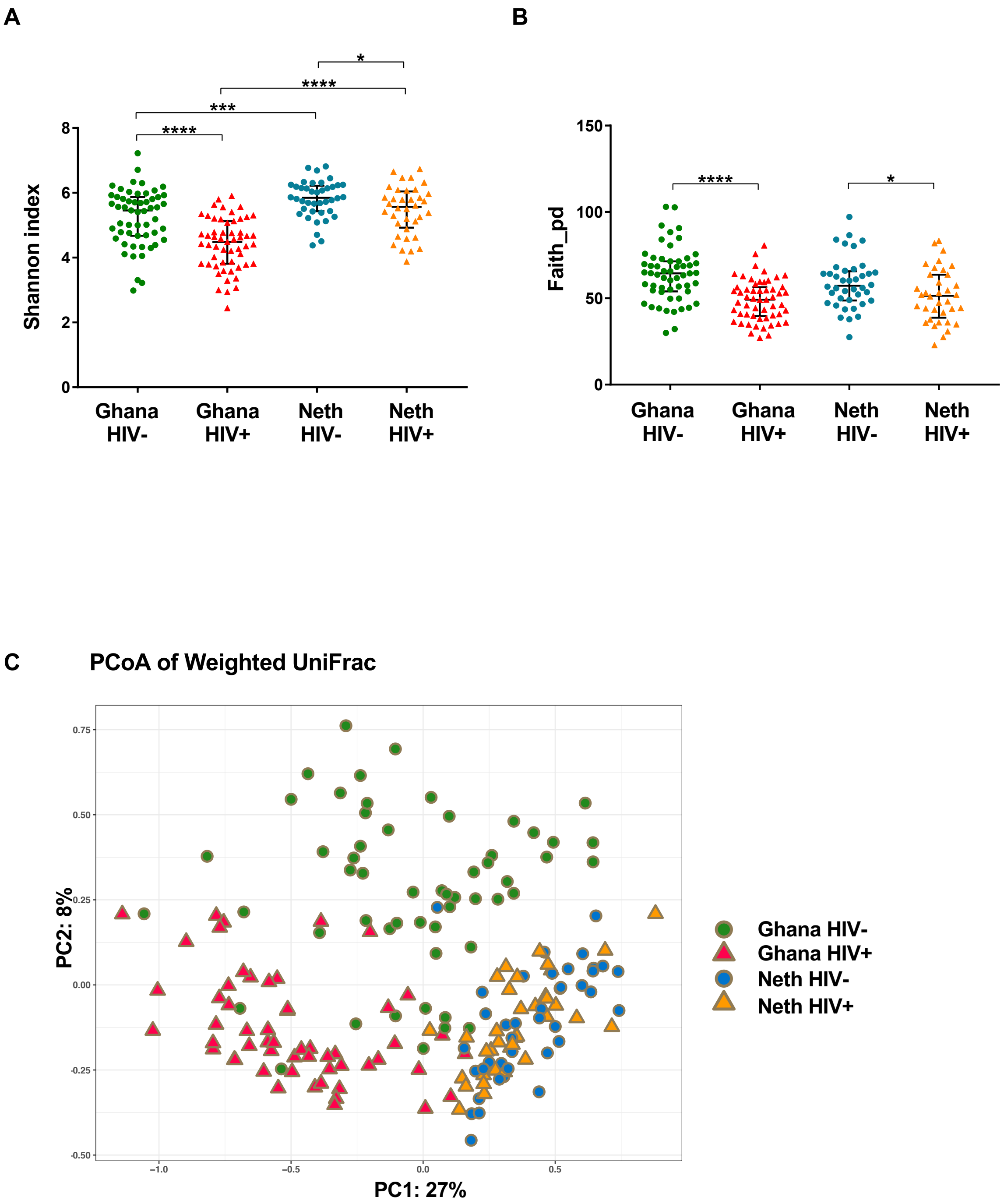

Supplementary Figure 10. Comparison of fecal microbiome diversity in the present study with a cohort from the Netherlands.

(A) Comparison of fecal microbiome Shannon diversity among HIV-1 uninfected individuals in Ghana (Ghana HIV-; n = 55), HIV-1 infected individuals in Ghana (Ghana HIV+; n =55), HIV-1 uninfected individuals in the Netherlands cohort (Neth HIV-; n = 40), and HIV-1 infected individuals in the Netherlands study (Neth HIV+; n =36). **(B)** Comparison of Faith phylogenetic diversity among Ghana HIV-, Ghana HIV+, Neth HIV-, and Neth HIV+. **(C)** Comparison of PCoA of weighted-unifrac distances among Ghana HIV-, Ghana HIV+, Neth HIV-, and Neth HIV+. Significant difference was determined by KruskalWallis test with Benjamini, Krieger and Yekutieli FDR correction; *, **, ***, and **** indicate significant differences with $p < 0.05$, $p < 0.01$, $p < 0.005$, and $p < 0.001$, respectively. Data from none-MSM participants in the Dutch cohort including 37 males (HIV- = 20 , HIV+ = 17) and 39 females (HIV- = 20 , HIV+ = 19) were used. Accession number PRJNA589036 (BioProject).
