# Supplementary material for: Dysbiotic Fecal Microbiome in HIV-1 Infected Individuals in Ghana
Source: Front Cell Infect Microbiol. 2021 May 18;11:646467. doi: 10.3389/fcimb.2021.646467 (PMC8168436; doi:10.3389/fcimb.2021.646467)
Supplement: Supplementary file 2 [file DataSheet_2.pdf]

Supplementary Information 1
Map of the eastern region of Ghana showing the study communities

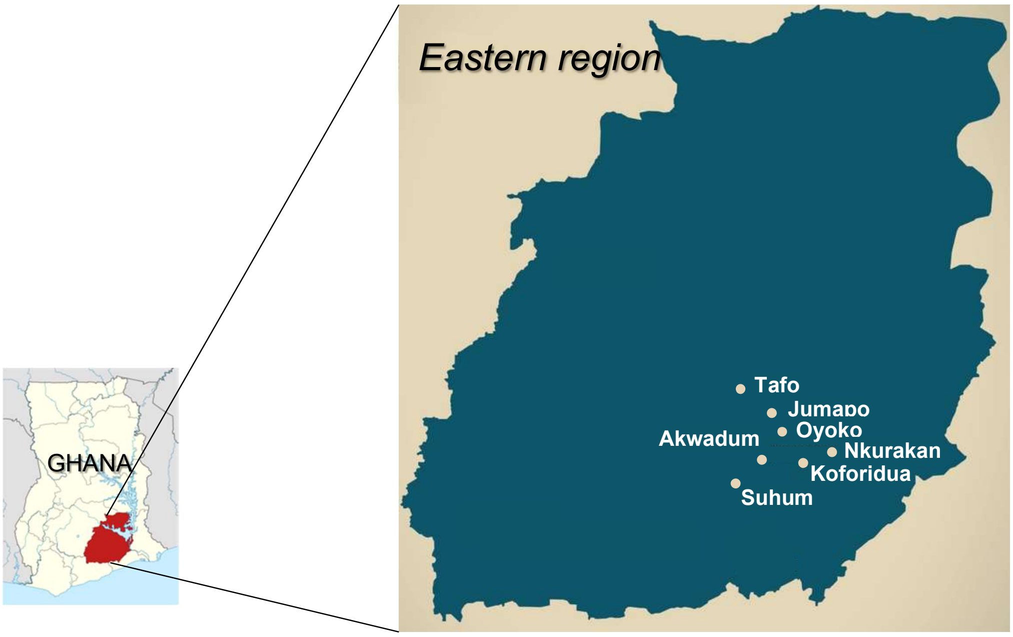

Study participants resided in 7 communities located within an area of 1200 km^2 in the eastern region of Ghana. The eastern region has a land area size of 19,323 km^2 with an estimated population density of 152.8 per km^2 during the study period*. The study communities are linked with 3 ~ 28 km of distance between community pairs. About 57% of the population in the eastern region reside in rural communities with agriculture being the predominant economic activity*. However, our study communities were mostly located at an interface of urban and rural areas, referred to as “peri-urban” communities, and 49% of our study participants were traders/merchants with only 7% being farmers. The top cause of morbidity in the region was malaria, while detailed information on health status of seronegative controls was not available in the present study.

*References:
Ghana statistical service (2021). 2010 population and housing census. Regional analytical report, Eastern region.
https://www.ghanaids.gov.gh/mcadmin/Uploads/2017-2022_national_and_sub%20national_Estimates_Report(1).pdf
(Date accessed: March 24, 2021).

Eastern Regional Co-ordinating Council (2021). Profile.
http://www.easternregion.gov.gh/index.php/profile/ (Date accessed March 24, 2021).

Supplementary Information 2
Sequence data analysis parameters (QIIME 2™ version 2019.4)

Prior to analysis with Qiime2, 5' adapters and primer sequences were removed with Cutadapt v1.18 maintaining default settings. (https://cutadapt.readthedocs.io/en/v1.8/guide.html).

1. Demultiplex/Import

```
qiime tools import
  --type 'SampleData[PairedEndSequencesWithQuality]'
  --input-path pe-33-manifest
  --input-format PairedEndFastqManifestPhred33V2
  --output-path demux_output.qza
```

2. Denoising

```
qiime dada2 denoise-paired
  --i-demultiplexed-seqs demux_output.qza
  --p-trim-left-f 13
  --p-trim-left-r 13
  --p-trunc-len-f 270
  --p-trunc-len-r 180
   --output-dir denoise_output_directory
```

3. Training feature classifiers/ Taxonomic analysis: Reference 16S rRNA sequences from SILVA database (release 132) were trimmed to the V3-V4 region and used in classifying sequences using the Naive Bayesian classifier

```
qiime feature-classifier extract-reads
  --i-sequences 85_otus.qza
  --p-f-primer GTGCCAGCMGCCGCGGTAA
  --p-r-primer GGACTACHVGGGTWTCTAAT
  --o-reads extracted_reads.qza

qiime feature-classifier fit-classifier-naive-bayes
  --i-reference-reads extracted_reads.qza
  --i-reference-taxonomy ref-taxonomy.qza
  --o-classifier SILVA_V3_V4_classifier.qza

qiime feature-classifier classify-sklearn
  --i-classifier SILVA_V3_V4_classifier.qza
  --i-reads denoise_output_directory/rep-seqs.qza
  --o-classification sample_taxonomy.qza
```

4. Diversity analysis: The least sequence count for samples was 35564. Hence diversity analysis was performed on samples rarefied at 30000 sequence count. All other parameters were set at default.

```
qiime phylogeny align-to-tree-mafft-fasttree
  --i-sequences denoise_output_directory/rep-seqs.qza
  --output-dir Phylogeny_output_directory

qiime diversity core-metrics-phylogenetic
  --i-phylogeny Phylogeny_output_directory /rooted-tree.qza
```

```
--i-table denoise_output_directory/table.qza
--p-sampling-depth 30000
--m-metadata-file sample-metadata.tsv
--output-dir core-metrics-results
```

5. Data were then exported from qiime2 to perform statistical analyses and generate graphs and figures using GraphPad Prism version 7.04 and R v3.6.0 packages. Differentially abundant taxa by HIV-1 status were identified using the analysis of composition of microbiome (ANCOM) and linear discriminant analysis (LDA) effect size (LEfSe) methods (Segata et al., 2011; Mandal et al., 2015).
